# A qualitative study of the factors influencing recruitment to a pilot trial on the prevention of striae gravidarum

**DOI:** 10.1186/s12884-020-2781-x

**Published:** 2020-02-12

**Authors:** Miriam Brennan, Mike Clarke, Declan Devane, Maura Dowling

**Affiliations:** 1 School of Nursing and Midwifery, Aras Moyola, National University of Ireland Galway, Galway, H91 TK33 Ireland; 2 Centre for Public Health, School of Medicine, Dentistry and Biomedical Sciences, Institute of Clinical Sciences, Block B, Queen’s University Belfast, Royal Hospital, Grosvenor Road, Belfast, BT12 6BA Northern Ireland

**Keywords:** Pregnancy, Striae gravidarum, Stretch marks in pregnancy, Trial participation, Qualitative research

## Abstract

**Background:**

Striae gravidarum are a common occurrence in pregnancy and many women use a topical product to prevent their development or lessen their appearance if they do develop. There is a lack of evidence on the effectiveness of many of the products used by women. This study arose from challenges in recruitment to a pilot randomised trial (ISRCTN trial registration number:76992326) designed to evaluate the feasibility of a definitive trial to compare a moisturising oil to no treatment in the prevention and reduction in severity of striae gravidarum. The study reported here explored the factors influencing recruitment to that pilot trial.

**Methods:**

A qualitative descriptive study was undertaken involving primigravid women attending an Irish maternity hospital. Data were collected by semi-structured telephone interviews over a four-week period and analysed using the framework method of analysis. Fifteen interview transcripts were included in the analysis.

**Results:**

Four main themes consisting of twelve categories were identified from the interview data. The themes focused on women’s prevention of stretch marks and their choice of anti-stretch mark product, who and what influenced that choice and influences on trial participation. In relation to influences on trial participation, the possibility of being randomised to the non- intervention or control group was a deterrent for many women.

**Conclusions:**

The prevention of stretch marks is important to pregnant women, as is their choice of product to prevent them. Offering women the opportunity to be part of a trial that would be of low burden and would test a well-known product may optimise recruitment. However, reluctance to be randomised because of the possibility of being allocated to the non-intervention control group suggests that further work is needed in this field on how best to communicate uncertainty to potential participants.

## Background

Striae gravidarum or stretch marks are common in pregnancy, usually during the third trimester. They have been shown to affect 50 to 90% of pregnant women [1] and commonly occur during their first pregnancy [2]. Striae gravidarum frequently occur on the abdomen but are also seen on the breasts and thighs [3]. They appear as reddish or purple streaks and remain as glistening lines on the skin [3] and have been more recently described as ‘...scar like, hypopigmented linear bands displaying a crinkled shiny surface...’ [4] (p.750).

The cause of striae remains unclear but they may be related to the effects of stretching or tension on the dermal extracellular matrix involving the elastin and collagen elements. According to Shuster [5] (p.161) they are always associated with stretching and occur in skin where ‘...the connective tissue is partially mature with a critical titre of rigid cross linked collagen and ‘elastic’ unlinked collagen...’. Others report that there may also be some changes in the elastin fibre network important in skin elasticity, as a result of persistent strain on the dermal tissue, which may be related to a deficiency in cutaneous fibrillin [6]. Even moderate strain may be enough to damage the elastic fibre network [6]. More recent research suggests that there may be inadequate collagen repair which has been disturbed by skin stretching [4].

Several risk factors are associated with striae but not consistently so [7]. Risk factors can be classified as maternal factors in place before pregnancy, for example, family history of striae or young age, maternal factors during pregnancy, for example, increased weight gain and increased body mass index at birth of baby and neonatal factors, which include increased gestational age at birth of baby and increased birth weight [7]. Pregnant women with one or more of the ‘attributes’ may be at increased risk for striae gravidarum [7] (p.607). In a recent study addressing risk factors for striae in primigravid women, Kocaöz et al. [8] found increased risk in those without social security, who sleep nine or more hours per day, have a body mass index (BMI) of 30 kg/m^2^ or more and have a family history of striae.

Striae can have both physical and psychological implications for women. They can be seen as disfiguring [9, 10] and are regarded as a significant cosmetic issue [11]. They can impact on a woman’s perception of herself [12], and cause ‘psychological distress’ [13] (p.595). Furthermore, although not impacting on general quality of life, striae have been found to impact women’s dermatology specific quality of life based on scores obtained on the emotion scale of Skindex-29 used by Yamaguchi et al., [14]. More recently, Kocaöz et al., [8] report how women’s body image declined in the presence of striae and their increasing severity. While acknowledging that striae gravidarum can impact negatively on many women, some women have a different experience and accept them as a normal part of pregnancy, as reported by Yamaguchi et al., [14]. However this latter perspective is just one single report and the negative aspects of striae tend to be the main focus in the literature.

That striae are of significant concern to women of child bearing age is reflected in the use of products for their prevention or to reduce their severity. In our large survey of 753 pregnant women in Ireland [15], most respondents (78.2%, *n* = 589) indicated that they used a product to prevent or reduce the development of stretch marks during their current pregnancy and more than one third (36.5%, *n* = 210) had used two or more products. This was similar to a Japanese study [13] but higher than other earlier studies [1]. Most recently, 40.9% (*n* = 172) of participants in a Turkish study reported using a product [8].

However, studies addressing product effectiveness are few in number [7]. A Cochrane Review [16] found no high-quality evidence to support the use of any of the topical preparations identified in the review for the prevention of stretch marks during pregnancy. Furthermore, the review recommended that preparations commonly used by women to prevent and treat stretch marks should be evaluated in large trials [16]. To address this, we developed a protocol for a pilot randomised trial (ISRCTN: 76992326) to evaluate the feasibility of conducting a definitive trial to evaluate the effectiveness of a moisturising oil (Baby oil) compared to no treatment in the prevention and reduction in severity of striae gravidarum. Following ethical approval, the pilot study sought to recruit women between 12 and 14 weeks’ gestation attending for booking at the antenatal clinic of the maternity unit in an Irish hospital. However, recruitment was challenging and as we were unable to recruit women to the trial, following further ethical approval, it was decided to adjust the gestation inclusion criterion from 12 to 14 weeks to 12–16 weeks to maximise the chances of recruitment. Nevertheless, despite this adjustment and a concerted effort to recruit women during 4 weeks in February 2018, it was evident that recruitment may not be possible. Challenges with recruitment are not unique to this study. Challenges with recruitment to trials in maternity care are reported by others [17–20]. In light of this, and following a review of the literature on maternal trials and discussion with maternity staff, we decided that an exploration of the factors influencing recruitment to the trial was necessary to elucidate factors influencing recruitment and to guide future research on this topic. Efforts to recruit to the trial continued following ethical approval and women who declined to participate in the pilot trial were invited to participate in a qualitative study to explore the factors influencing recruitment.

## Methods

### Study aim

To explore factors influencing recruitment of women to a pilot trial on the prevention of striae gravidarum.

### Study design, setting and participants

The design adopted was qualitative descriptive [21, 22]. Qualitative research is a means of investigating how people understand their experiences and their world [23]. Therefore, this approach enabled a close interaction with purposively chosen participants to understand in more depth the factors influencing recruitment to the pilot trial on the prevention of stretch marks in pregnancy. Furthermore, while there is a growing body of qualitative evidence in relation to influences on trial participation by pregnant women, we are not aware of any study which focused specifically on stretch mark prevention in pregnancy.

In relation to sample size, the research was guided by the work of Sandelowski [21] who advises that the research team must make a judgment on what is an adequate sample size. Morse [24] also highlights how many factors influence the sample size required for data saturation in qualitative studies, including the study scope, ‘nature of the topic’ and the quality of the data. In light of these factors, it was proposed to interview 10–15 participants depending on the point at which data saturation is reached, i.e. no new data is being obtained and data is being repeated by participants [25].

Eligible study participants were English speaking primigravid women with a singleton pregnancy, aged 18 or over, attending their first visit (booking appointment) in the antenatal clinic of the maternity unit in an Irish hospital who had declined to participate in the pilot trial outlined above. On their first visit (booking visit), eligible participants were approached by a researcher (MB) and informed of the pilot trial. When a woman declined to participate in the pilot trial, she was informed of this qualitative study and offered the information pack, which included a cover letter, participant information sheet, consent form, the interview guide and a stamped addressed envelope.

Participants who took the information pack were asked if they were agreeable to be contacted by telephone after 24 h to address any questions they may have and to ascertain if they were willing to participate in a short semi-structured telephone interview, lasting 20–30 min at a mutually acceptable time. Thirty-five women agreed to the follow up call.

Following the call, 16 primigravid women between 12 and 16 weeks’ gestation agreed to take part in semi-structured telephone interviews exploring the factors influencing recruitment to the pilot trial on the prevention of striae gravidarum. Interviews were audio recorded, took place over 4 weeks in June and July 2018 and lasted between eight and nineteen minutes each. Two women provided written consent before their telephone interviews. The remainder returned the consent form following their interview. However, one participant did not return the completed consent form despite a follow up reminder and therefore her interview data were not used in the analysis.

While telephone interviews can be challenging in establishing a rapport, MB had met all the study participants in the antenatal clinic at recruitment and also spoke to them during the follow up telephone call to arrange the interviews, so a rapport had already been developed before the interviews.

### Reflexivity and rigour

In cognisance of the role of the researcher in qualitative research [26] and the importance of reflexivity in the process [25, 27], MB was aware of potential biases that could impact the study at the different stages but in particular during data collection and interpretation. Possible biases included MB’s prior assumptions about the importance of striae prevention to pregnant women, and reasons for women not agreeing to participate in the trial as realised during the recruitment effort. While it is almost impossible to avoid having some views on what may emerge during analysis, the researcher must strive to ensure that the participants’ views are truly represented [23]. Recognition of these potential biases was important in light of the close relationship MB had with the topic under investigation over an extended study time period where MB had previously undertook a Cochrane systematic review and meta-analysis [16] and a descriptive cross-sectional survey on the topic [15].

In relation to the data collection process, MB considered the interview aim and required outcome [25], in addition to the need to have a trusting relationship with participants towards meeting the study aim and in particular accessing their views on the topic. MB was aware from earlier research that striae gravidarum are more important to some women than others and the need to hear and reflect both perspectives. During and after the data collection it was evident that the first encounter in the antenatal clinic was crucial to building a good relationship and was commented on by some of the participants towards the end of the interview process. Throughout the interview process, MB aimed for ‘empathic neutrality’ [26] while also relaying an interest [25] in the woman and her pregnancy.

We found that the framework method of analysis supported the maintenance of rigour, in particular the credibility or the ‘...truth of the data and the interpretation of them’ [28] (p.585), the confirmability and also the transferability of the data.

### Data collection and analysis

An interview guide developed by all authors was used and only minor changes were made following the initial interviews. Each interview began with an open question on the reasons for not participating in the pilot trial. Thereafter, the interview followed a semi-structured format using the topic guide [29], which included questions around product choice, influences on product choice, confidence in effectiveness of chosen product, and influences of randomisation and specific trial requirements (such as diary keeping) on the decision to not participate in the pilot trial. The topic guide was applied flexibly in response to the participants’ responses. This method offered flexibility for the participants and facilitated them in sharing their perspective in their own words. Data saturation was evident by the tenth interview. As MB is a novice qualitative researcher, data collection and analysis were overseen by MD who is an experienced qualitative researcher.

Framework analysis [30] guided the analysis process. It is a process developed in the 1980s in the UK by social policy researchers Liz Spenser and Jane Ritchie (Spenser & Ritchie 1994). This analysis process entails clearly defined stages that are closely interrelated and requires the analyst to systematically sift, chart, and sort the data in relation to major topics and themes. While offering a methodical way to undertake analysis, it also depends on the inventive aptitude of the analyst to be able to see the connotations, significance and interrelationships in the data [30]. It can be used flexibly and fitted to the needs of an individual study. This approach belongs to an expansive group of methods of analysis often referred to as thematic analysis or qualitative content analysis [31]. The Framework method of analysis was considered a suitable method for the inductive thematic analysis of semi-structured interview transcripts, which were used in this study and which were consistent with the study aim [31]. Moreover, data were homogenous and suitable for classification, which is a requirement of the Framework method [31]. Management of the data involved the following five steps:

#### Step 1 Familiarisation

Firstly, transcript auditing [32] was undertaken by MB to check for accuracy as interviews were transcribed professionally. All the transcribed interviews were read several times to get an overview of the content and to become more familiar with it. During this stage, MB got a comprehensive understanding of the data, identified items of interest and recurrent topics or themes relevant to the study aim and compiled a comprehensive ‘inventory’ [33]. Following this, as required by the method, comprehensiveness of the inventory was verified by checking it against the interview guide, while each item on the inventory was checked for relevancy against the study aim, resulting in a broad list of topics or themes present in the data.

#### Step 2 Construction of the initial thematic framework

This second stage involved grouping and sorting the themes to form a hierarchical structure of themes and sub themes [33, 34], which were labelled with descriptive terms staying ‘...close to the language and terms used in the data set’ [34] (p.222). This stage resulted in ten themes with varying numbers of sub themes (total-39), including an ‘other’ category in some themes which were reclassified subsequently. Further revision resulted in eight themes and 25 sub themes (See eight themes in Table 1).

**Table 1:** Framework with themes from the end of step 2, step 5, and with final themes

End of step 2	Step 5	Final
1. Anti-stretch mark product chosen	1. Prevention of stretch marks and anti-stretch mark product choice	1. Preventing stretch marks and choice of anti-stretch mark product
2. Influences on decision or choice of product	2. Influences on product choice	2. Who knows best?
3. Confidence in chosen/planned product	3. Influences on trial participation or not	3. Influencers: Current trial participation
4. Influences on trial participation	4. Influences on future trial participation	4. Influencers: Future trial participation
5. Influences on future trial participation		
6. Influences on future anti- stretch mark product purchases		
7. Stretch marks in pregnancy		
8.Other		

#### Step 3 Use of the framework for indexing and sorting the data

MB then cross checked each transcript with the framework, identifying where each topic appeared and labelling it with the framework or thematic reference, which Spencer et al. [33] refer to as ‘indexing’. Following indexing, a table was devised for each theme and sub theme, which also brought together data of similar content and indexed it accordingly for each participant or case.

#### Step 4 Reviewing data extracts

Further refinement of the framework involved reviewing the indexed data and looking back over the transcripts again to detect any omissions in indexing or from the framework. This resulted in some changes to titles of themes and sub themes, and some sub themes were merged to reduce fragmentation of the data. This necessitated some re-labelling and changes to indexed numbers. Memos were kept of all changes made at each stage. The framework at this stage consisted of six themes and 23 categories.

#### Step 5 Data summary and display

This stage involved the development of framework matrices for each individual theme, which is specific to framework analysis. Matrices are constructed in such a way that comparisons can be seen between different parts of the framework at the individual level and ‘...across cases within a single thematic matrix’ [33] (p.305). MD reviewed the matrices in conjunction with reading all 15 transcripts and following discussions with MB, further refinement of the framework occurred with minor changes being made to some wording and moving and collapsing of some sub themes (Four themes and 15 sub themes) (See four themes in Table 1).

### Abstraction and interpretation

These five steps were followed by further discussions between MB and MD and identification of constituent elements and underlying dimensions, identifying possible categories and finally group classification or themes [33] to reflect the data. This entailed trying to move from ‘surface features of the data to more analytic properties’ [33] (p.285). It also involved some refinement and movement of sub themes (now referred to as categories) within the framework (See final themes in Table 1).

In terms of rigour, notes were kept at all stages of the framework analysis method, providing an audit trail which contributes towards dependability of the data. Furthermore, all stages of the analysis were validated by MD in support of confirmability of findings. Finally, the framework method with its systematic and comprehensive data analysis, facilitates both within and between case examination and transparency [33].

## Results

After analysis, four main themes and 12 categories were identified from the interview data (Table 2) on factors influencing recruitment to the pilot trial on the prevention of striae gravidarum.

**Table 2:** Final themes and categories identified

Preventing stretch marks and choice of anti-stretch mark product	• Better to try prevent than do nothing
	• Knowing or not knowing what product I will use
	• Prior knowledge
	• Keep it natural
	• Will it work?
	• Cost
Who knows best?	• Friends and Family
	• Retail and Advertising
Influencers: Current trial participation	• I want a choice
	• Trial requirements should fit with my routine
Influencers: Future trial participation	• I want a good quality product with a good name
	• Incentives may be helpful

### Preventing stretch marks and choice of anti-stretch mark product

Stretch marks and their prevention during pregnancy was important to many of the participants and they had already thought about and planned to use a topical product to try to prevent the development of stretch marks during their pregnancy. Many had actually purchased their chosen product and, in some cases, had started to use it.

For many of the women, there was an innate belief that they were better to apply a product to the skin during pregnancy to try to prevent stretch marks rather than not applying anything. For some, it was the moisturising or hydration aspect, while for others they were ‘taking no chances’ and viewed any product as potentially beneficial. Some products were more popular than others; specifically, many women wanted a ‘natural’ product and emphasised the organic nature of the product.

Cost was a factor in product choice for many participants. For others, cost was not an important factor but they felt it may have become one if they were actually getting stretch marks. Where cost was important in the choice of product for some participants, it was related to unknown effectiveness in that they did not wish to spend a lot of money on anti-stretch mark products particularly as they did not know if they would work. Others always went for a cheaper product. Many of the participants had a cut-off point or a price that they would not go above. Other participants were going to buy their product of choice whatever the cost and would pay what was required (Table 3).

**Table 3:** Themes and categories illustrating women’s views in response to questions relating to preventing stretch marks and choice of anti-stretch mark product

Theme	Categories	Sample Quote
Preventing stretch marks and choice of anti-stretch mark product	Better to try prevent than do nothing	*I’m hopeful it will* [work] *but I suppose I’m thinking I’m probably in a better position by using it than not doing anything.* […] *I’d rather be trying to prevent them than just doing nothing.* (Participant 1) *I just know it’s important to moisturize. (*Participant 3) *… so I think it would be great to just hydrate it and keep the skin a little bit more hydrated.* (Participant 4) *I am probably 70 % confident that it will help me but I don’t know if anything can prevent them you know.* (Participant 6) *...I think that it if you are going to get stretch marks you are probably just going to get them anyway but I think that maybe it just might help to decrease them.* (Participant 8) … *definitely I would feel like you are better off using a rich moisturising cream than using anything at all* […] *I would prefer to definitely do something on myself because I feel like a little bit of technique and creams or whatever it may be you know even if it is actually [product name] that would be better than nothing.* (Participant 10) *...I frankly don’t want stretch marks*...(Participant 12) *...I suffer from stretch marks through puberty* […] *I will try and prevent it now if possible and so I gave I mean I would give it a go by applying cream*. (Participant 13) *...I had picked a product it was like reasonably cheap and I suppose I have stretch marks already on my legs and stuff so I would be prone to them anyway but it was kind of one of my reasons just like if I don’t want to get them* […] *if you can prevent them I suppose if you don’t want something for like even though they have fade down they are still you are still scared like do you know.* (Participant 14) *… I am just not a risk taker so I would want to use a product no matter what*. (Participant 16)
	Knowing or not knowing what product I will use	*I had already started using a cream by the time I met you* [researcher] *in the hospital.* […] *it’s a spur of the moment* […] *so I threw it in the basket, there was no, you know, intention beforehand or anything* […] *it was literally only that day that I saw the cream and just thought oh sure I’ll throw this in the basket*. (Participant 1) *I had planned on using a product myself* […] *I had my own kind of idea of what I wanted to do already at that stage* […] *what I’m using at the moment is just the [product name] on myself but I’m going to use [product name] …* (Participant 2) *… I actually haven’t even thought about it until you* [researcher] *spoke to me that day...* (Participant 5) *I am about the fourteen weeks and I haven’t any stretch marks yet I thought it is too soon but I am already beginning to apply cream just in case.* (Participant 6) *...I have kind of had it in my head to use that [product name].* (Participant 8) *Right now it’s not a priority for me I am not even thinking about stretch marks and I don’t think it would be I probably won’t use anything. I am certainly not planning using anything from or at this stage …* (Participant 9) *...I have chosen to use [product name]* […] *I didn’t have to go out and buy it, it was something that I had before I became pregnant so it was easy access in that I already had the product.* (Participant 11) *...I am currently applying [product name] to try and prevent stretch marks*. (Participant 13) *...I would use [product name] for moisturizer* […] *Well I had extremely dry skin there for a while and I just thought that that would be good for dry skin so it wasn’t really for the stretch marks but means I have the bottle now I may as well use it.* (Participant 14) *...I actually decided I came to the conclusion that I will be going for [product name] … (*Participant 15)
	Prior knowledge	*...I’ve used the same face cream probably for the last fifteen years I’d probably look to that range first as well to see do they have a product available for body moisturising especially for stretch marks* […] *if I had heard it had good reviews about it I certainly would try it.* (Participant 3) *… like clinical trials and attempts that were done in this university. They wrote articles and I followed that for a good while you see that is 2015, 2016 really I wasn’t pregnant but I was doing an awful lot of reading about it.* (Participant 10). … *I would have looked at ingredients in each of the products …* (Participant 13) ..*none of them are proven they don’t state that they are going to eliminate it forever you know so I would always go for a cheaper option. But I still like a brand name like I like the branding and I like the good quality [product name].* (Participant 14) *...I did a bit of research myself but at the same time my main priority was to use a natural product as natural as possible.* (Participant 15)
	Keep it natural	*...I’d like something a little bit more organic, rather than, I got the [product name]* […] *But I found it very kind of chemical smell off it. I wasn’t that into it. So my other sister was saying about some sort of cream from the health shop that was more organic.* (Participant 4) *...I love anything that is natural I am a vegetarian I am very much against any animal testing or animal cruelty. So those factors would be hugely important to me.* (Participant 6) … *it’s* [product name] a *nice smelling kind of oil and also just that it actually says on the [product] that it does prevent stretch marks or minimises the appearance of them.* (Participant 8) *...I can’t remember the exact name of it but it’s basically the option that you had for stretch marks that natural skin care company[…*] *Basically they are all natural because I don’t use anything that has got preservatives in it so I also have sensitive skin*. (Participant 10) *...I would use [product name] for moisturiser* […] *I find it very nice and a nice smell and it’s kind of leaves the skin very soft.* (Participant 14) … *it’s a pure essence basically an orange oil that actually helps with stretch marks. And it is anti-oxidant as well so it’s good for everything* … (Participant 15)
	Will it work?	*...I’m hopeful it will* [work] … (Participant 1) *...I honestly don’t know how it’s going to work …* (Participant 2) *I’m not too sure if it’s* (pauses) *a particular product or a particular* (pauses) *oil* versus *cream, I just know you should moisturize.* (Participant 3) … *not 100%,* (laughing) *hmm* (pauses) *I don’t know, I won’t know.* […] *...I do think it is hereditary as well. Both of them* [sisters] *no one in my family seems to, touch wood, to get stretch marks.* (Participant 4) *...I suppose my belief I don’t have any proven findings of fact. I mean a lot of these are research and you know it may work for some women it may not but I would hope that it would work for me*. (Participant 10). *...I don’t know how confident I am that it will work …* (Participant 11) *...I am probably not very confident I am probably aware of the fact that I am predisposed to getting them anyway it might it might help* [pauses] *I suppose maybe heal them faster by going from you know from the red you know the initial ones that they are red or purple and move to silver. I suppose maybe that is what I am trying to do to get give me make my skin as prepared as possible.* (Participant 13) *...I suppose I wouldn’t be that confident because I suppose I just think that you are going to get them you will probably get the stretch marks I don’t know can you fully prevent them.* (Participant 14) *But I am not obviously a hundred per cent sure that it will work just it was just recommended this one from this people that helped me to make my choice and to trust that product.* (Participant 15) … *maybe you are going to get stretch marks no matter what you use* […] *other people saying it doesn’t matter what products you use you know stretch marks are genetic so you are going to get them if you are going to get them you are going to get them no matter what.* (Participant 16)
	Cost	*I think it was about five or six euro like to me that didn’t seem that expensive it’s a big enough bottle.* (Participant 1) *… if I was getting the stretch marks I don’t think it* [cost] *would be huge, a huge factor in it* [choice of product]. (Participant 2) *I mean it* [cost] *would certainly play a part …* (Participant 3) *But I wouldn’t go spending like 50 euro on one bottle of cream now. But hmm (pauses) yeah, it wouldn’t influence me too, I wouldn’t go too high a price bracket but yeah, there’s no point in getting something really cheap just for the sake of it either.* (Participant 4) *… I wouldn’t be spending a huge amount of money on anything like that.* (Participant 5) *I would be a middle of the range price person […*] *I wouldn’t buy anything maybe fifty euro or more*. (Participant 6) … *like I think if you feel something is going to work it doesn’t really matter how expensive it is I mean I well actually [product name] isn’t that expensive hmm you know I don’t know I suppose you wouldn’t want to be spending too much either. I mean yes it has to be kind of a reasonable price* … (Participant 8 *...I would be within a reasonable price point I am not going to go up to eighteen or even sixteen euro on something that may or may not work you know something that is going to be a natural product is not going to cost the earth anyway because it wouldn’t be made by a huge big brand.* (Participant 10) … *cost wouldn’t have influenced my decision in picking a product over the other.* (Participant 11) *… it was very expensive for a small bottle I think it was twenty something euro but like I just like I suppose it will probably last a good while and if it stops the stretch marks I don’t really mind how much it’s going to cost.* (Participant 12) *… yes I wouldn’t want to be spending a fortune on something that I am not a hundred per cent will work anyway.* (Participant 13) *… I would always go for a cheaper version … (*Participant 14) *So I actually didn’t even look into the prices just about the recommendations and then the price* […] *I would not look into [price] I would pay what I would need to pay for it.* (Participant 15) *… the price is important. Like I mean if this bottle of [product name] is two euro at Ever Green that is nice but I would spend more than that on a product that I think is going to be high quality but not too much.* (Participant 16)

### Who knows best?

This theme captures who or what resource was accessed most frequently by participants. Friends and family were a key influence on choice of anti-stretch mark product. Friends and particularly those who had been pregnant previously were often reported by participants as a source of influence. Participants often added that their friends had recommended the product and or had found it effective and participants seemed to value the advice and recommendations they received in most cases. Other sources also influenced women’s choice. Some noticed products when shopping, while others were more targeted with some women referring to a local health food store and how they talked to the assistants there (Table 4).

**Table 4:** Themes and categories illustrating women’s views in response to questions relating to resources accessed in choosing an anti-stretch mark product

Theme	Categories	Sample Quote
Who knows best?	Friends and Family	*… recommendations from friends who have been pregnant before …* (Participant 2) … *one of my sisters used the [product name] and she found it great.* (Participant 4) *… I have a couple of friends that are nurses and I would probably ask them as well if they used anything or they found anything good.* (Participant 5) *...I purchased it* [product name] *as a gift from my good friend who became pregnant last year and she swore by it. She said it was fantastic so based on her recommendation...* (Participant 6) *I have a family member who have used it before and she didn’t get any stretch marks and she felt that it worked really well*. (Participant 8) … *I had heard friends say that they used it* […] *and they found it effective.* (Participant 11) *...I am only going with what people told me so* [...] *family and friends telling me to apply the [product name] to the stomach.* (Participant 12) *… my friends one of my friends recommended the [product name] [pause] stretch mark cream and I think that is kind of one just one girl recommended that but I didn’t get it because I had the other one already. I have the other ones already got … (Participant 14)*
	Retail and Advertising	*… just from generally from shopping and seeing what’s on the shelves.* (Participant 3) *I was going to go into Ever Green* [Health food store] *and ask them what they thought.* (Participant 4) *...I would kind of do a bit of research just through the internet just to see on the maybe on the forums and that what people use and use that sound good.* (Participant 5) *...I have seen ads on television for the oil as well.* (Participant 8) *… advertisement of [product name] that it advertises that it helps like in prevention of stretch marks and maybe why I picked it as well.* (Participant 11) … *it’s the pharmacist advised that I do it for no stretch marks … (*Participant 12) *...I am* […] *big into natural products and I kind of I went to the Evergreen shop* [Health food store] *and I discussed that with the health care professionals there.* (Participant 15) *...I have just started researching just at online reviews … (*Participant 16)

### Influencers: current trial participation

This third theme addresses the factors influencing participants’ decisions not to participate in the pilot trial. Central to this theme is the trial design or methodology that involved participants being randomised to a group that applied an oil to their abdomen or to a group that did not apply any product (control group). Women were unhappy that they might be allocated to a group that would not apply any product. Many participants related how they had a clear preference for the intervention group if they were to participate. Such favouring of the trial’s intervention suggested that despite the absence of evidence of effectiveness, the intervention was perceived to be better and this belief moved potential participants from a position of individual equipoise or uncertainty. One participant said she was already concerned about stretch marks and would be put off by being in the group that was not applying a product (participant 1). Others wanted to use an anti-stretch mark product and did not want to be in the non-intervention group (Participant 2 & 16), while another participant indicated that she always moisturised her skin and would not be prepared to stop.

Trials often have certain requirements from participants and this category captures participants’ willingness or not to comply with the pilot trial requirements, such as daily showering before application of the oil and the keeping of a paper diary during the study to record the use of the oil application on a daily basis. For some, these requirements were not a problem and would fit with their daily routine (Participant 1, 3 & 11) while for others they did not fit (Participant 8 & 9). Fitting in with participants’ daily routine was important and emphasised by many participants.

Some participants’ responses related to not being able to commit to doing either the showering, completing the diary or both and they often identified implications of this for the overall study. Where this occurred, participants acknowledged openly the importance of adherence to the requirements for the overall study integrity. Examples include participant 2 who did not like the commitment to the trial requirements and mentioned about recording inaccurate detail in the diary. Similarly, participants 5 and 16 mentioned not having the dedication to comply and were aware of the impacts of this on the study results. Not being able to commit was also raised by participant 13, while for others, it was important that the requirements did not take up too much time (participant 14).

Some participants identified a preference for an on-line tool or other method of maintaining the diary on their telephone to fit their lifestyle (Participant 6). While others raised practical issues such as forgetting the diary if they were away at the weekend and suggested that a smart phone based system would work better for them (Participant 10) (Table 5).

**Table 5:** Themes and categories illustrating women’s views in response to questions relating to participating in the prevention of striae gravidarum trial

Theme	Categories	Sample Quote
Influencers: Current trial participation	I want a choice	*… if I was participating in it I think I’d prefer to be in the group that would be doing it as opposed to the group that’s doing nothing* […] *like if I was told I was in the group to do nothing well maybe I’d already been you know a bit concerned about stretch marks and so I’d be a bit more concerned you know maybe it would put me off.* (Participant 1) … *if it was randomised and then you were in the group that wasn’t that would kind of, that would have been one of the reasons as well for saying not to on it* […] *because I had it in my head I had planned on starting it I didn’t want to be randomised into the group that was not.* (Participant 2) *... if I was put in the group that wasn’t to use any moisturiser that straight away you know I’d only be lying* (laughing) *I’m not going to stop the habit of a lifetime.* (Participant 3) *...I think when the choices are taken out of your hand that is hardier for me. It was just picked at random I don’t know how you would pick it but I know I would struggle with random selection of how the people would be chosen.* (Participant 5) *If it was something that I hadn’t considered I wouldn’t mind being in either or of the groups obviously if it was no harm to myself or the baby there was no risk of harm.* (Participant 6) *...I think that if I did feel strongly one way or the other about whether I wanted to use the cream or not I wouldn’t mind taking part if I got to choose what options I would have to go with.* (Participant 9) *...I probably wouldn’t have been happy because I wouldn’t have had the choice you know*. (Participant 11) *...I would not want to be in the group without using any kind of product just because I feel like I would want to use one either way. Just to be as a precaution so if I was in one of the group without the oil and then I would (laughs) disappointed as I want to use something anyway.* (Participant 16)
	Trial requirements should fit with my routine	*...I shower twice a day and I’ve no problem I keep a diary anyway. So there would be no bother with either of those* [trial requirements]. (Participant 1) *...I wouldn’t like to commit to it and then forget to do it and then not, and put an inaccurate detail on it. But that would be a little thing actually for me when I think about it day to day.* (Participant 2) … *the cream would be my morning routine anyway* [*…*] *I wouldn’t have a problem with that* [diary], *you are not noting down, it wouldn’t take a massive amount of time.* (Participant 3) … *when you* [researcher] *gave me details of how the trial would be run and the amount of times you would have to apply it during the day. And that you would have to shower in the morning I just feel that I wouldn’t have the dedication to it to you know give you satisfactory results.* (Participant 5) *I would have no problem with that* [trial requirements] *if you use the online tool that I can keep track on my phone or something that would make it even handier for me.* (Participant 6) *… yes again that could be like people might just feel like it might get in the way of their routine kind of like maybe they don’t want to have a shower in the morning or they usually have their shower before they go to bed or something like that you know. That might be a problem or a nuisance or something.* (Participant 8) *...I can see how you need everybody to have the same conditions when they are applying the oil l can see why you set that kind of a standard. To be honest it’s just an extra piece of work I know like everybody showers of course but some people might be showering before bed and others might be showering in the morning. And if I need to do it every day and if they are leaving it a day and they can’t do that it’s another pressure.* (Participant 9). *I suppose if you keep it in a reminder on your phone excerpt for diaries because I know I am difficult to forget or to bring it with me or if I went away for the weekend you can forget your diary and where would you write it.* […] *If you had a little quick even a word like email google doc like you know you can get into on your phone all the time.* (Participant 10) *… that is a fairly easy because I suppose I leave the oil on my bedside locker so I could leave the pen and paper there as well and just jot it when I did it.* (Participant 11) *I don’t know if I would commit to that* [trial requirements] … (Participant 13) *...I suppose it would be fine if it was just like a tick like diary you know something like very quick.* […] *You don’t want things that take up too much* [time] … (Participant 14) *...I do know some people that don’t shower that often you know maybe once every three days or something so then I would think maybe it’s not if you weren’t showering as frequently then you are not applying the lotion every day. So and that would be the only issue I could see* … (Participant 16)

### Influencers: future trial participation

Participants also identified factors that would influence their participation in a future trial on stretch mark prevention in pregnancy. Although they were not keen on being randomised, they would be more inclined to join a study that involved the testing of a known anti-stretch mark product and often added that they would like to see some data or research to support the effectiveness of the proposed product. For others, the type of product was important with many women expressing a preference for use of an organic product or a natural product.

Participants also had views on the use of incentives in a future stretch mark prevention trial. Most participants would not be influenced to join the study unless they were already interested in participating in it. Incentives may be helpful but were not a major factor determining participation (Table 6).

**Table 6:** Themes and categories illustrating women’s views in response to questions relating to participating in future trials on anti- stretch mark products

Theme	Categories	Sample Quote
Influencers: Future trial participation	I want a good quality product with a good name	*I suppose it would be dependent on maybe reputation of it. Because I’ve heard [product name] is very good. Or I suppose if there was some study that showed that using that certain oil was seen to reduce the stretch marks by a certain percentage I suppose that would swing me towards using it*. (Participant 1) *I don’t actually really know if there was any, a moisturiser or that, that was effective* […] *I suppose research behind it.* (Participant 2) … *knowing the product and it being a product I would have heard of would probably gain my interest a little bit more.* (Participant 3) *Just something a little bit more organic and something that, yeah as I said absorbs into the skin, not a big film sitting on your skin. Yeah, just something kind of not too heavy …* (Participant 4) *...I would do research based on recommendations. That you know to find out if it’s actually as good as the price...* (Participant 6) *...I suppose if you have got trials if you had trials done already and you had a little bit of evidence that could support it …* (Participant 10) *...I suppose I would be more I would be swayed more by I suppose results or you know previous sort of you know if there was any if there was previous tests done.* (Participant 13)
	Incentives may be helpful	*Incentives like that* [money] *wouldn’t, no.* (Participant 2) *… I suppose a discount on the product.* (Participant 3) *...I suppose if I was a little bit more interested in it, it might persuade me if you got some little gift at the end of it.* (Participant 5 *… if there is a voucher at the end of it I wouldn’t turn it down either.* (Participant 6 *Yes I think an incentive like that be put in a draw or that you could win something that would definitely encourage people to be part of it.* (Participant 8)

Finally, participants were asked if they had been offered participation in other research studies after becoming pregnant (to determine if they might have been overburdened by research), but none of them reported any such offers and, therefore, it was not a factor in their decision to not participate in the pilot trial.

## Discussion

This qualitative descriptive study explored factors influencing recruitment to a pilot trial on the prevention of striae gravidarum, using semi-structured telephone interviews with 15 primigravid women. In keeping with a qualitative descriptive design and its ‘low-inference’ description [21], the findings have remained close to the original data and are relayed in common terms [21] through the themes and categories generated via the framework analysis method [33].

Our findings emphasise the importance of striae prevention for women during pregnancy. Many participants had already thought about stretch mark prevention at an early stage of pregnancy and had already planned to use a topical product to try to prevent them. This echoes previous research, where most respondents in a large survey in Ireland reported using a product to prevent or reduce stretch marks in pregnancy [15]. Many believed that they were better to apply a product to the skin during pregnancy (even it was not known to be effective) rather than not applying anything. They were not prepared to negotiate on anything that could increase their chance of getting stretch marks. This supports the views in the literature that many women are concerned by stretch marks [12], particularly because they do not completely disappear [35]. Moreover, although not a medical issue, these marks are a significant cosmetic issue for women [11, 36], commonly referred to as ‘disfiguring’ [7, 9, 10, 36]. Furthermore, they can cause distress to some women [2, 14]. Thus, women want to try to prevent them.

A range of anti-stretch mark products were chosen or in use by the participants, which concurs with other studies [1, 15]. Some products were more popular than others, which has also been identified previously [15]. Under the category ‘prior knowledge’, in the narratives we could see some of the influences on participants’ choice of product, for example, brand familiarity and perceived product quality. Natural or organic products were clearly important to some women. No evidence of these influences were uncovered in the literature. Women’s uncertainty on the relative effectiveness of their chosen product reflects the current status of knowledge on the effects of these products [7, 16, 36]. Finally, in relation to cost, some participants were prepared to spend more than others to get their product of choice. Although the amount of money spent by women is not widely reported in the literature, our earlier research showed that primigravid women are more likely to spend more money on anti-stretch mark products than multigravid women [15].

The influence of family and of advertising on participants’ choice of anti-stretch mark product is in keeping with our previous research, where 49.3% (*n* = 278) of respondents identified advice from friends in helping them choose a product, followed by 23% (*n* = 130) for product advertisement, 18.8% (*n* = 106) for advice from a family member and 14.7% (*n* = 83) from the internet [15].

The possibility of being randomised to the non-intervention or control group in the pilot trial was a significant barrier to participation and is reflected in the category ‘I want a choice’. Participants did not want to be allocated to the control group and not be able to use a product. Furthermore, some wanted to choose their own anti-stretch mark product. This suggests that some participants did not seem to understand trial design fully and this has been noted by others [20, 37]. Kenyon et al., [37] interviewed women about their experiences of being recruited to a trial on antibiotics in pre-term labour (ORACLE) and reported that some women seemed to have poor understanding of trial processes [37]. Similar points have also been made in non-maternity studies [38].

The issue that randomisation is a barrier to recruitment to research is reported by others. Ballantyne et al., [39] (p.480) report how some pregnant women when interviewed about their involvement in a randomised trial comparing a probiotic supplement versus a placebo (PiP study) in New Zealand, saw possible randomisation to a control group involving a placebo ‘as a burden or disadvantage’, although it was not the main barrier. However, they add how anecdotal evidence from staff revealed that one main reason for women not agreeing to participate in the trial was their wish to take a probiotic during pregnancy, which they would not have being able to do if randomised to the placebo group. While participants in our qualitative study were discouraged by the use of a non-intervention group, others have reported how women during pregnancy have said that they were less inclined to agree to placebo-controlled trials because of the uncertainty of receiving or not receiving an intervention considered advantageous [40]. Uncertainty around placebo use and randomised trials has also being identified by others [20, 41], and Rodger et al., [42] found that pregnant women identified fear of being randomised to the placebo group as a reason for non-participation. In addition, some participants in our study referred to lack of choice, which may be related to lack of control which was identified by some participants in a study which explored the factors influencing women’s decisions on participation in research during pregnancy [43]. Some women disliked the idea of randomisation due to its reliance on chance and were possibly discouraged from participating due to the chance element and lack of control with a trial design [43].

These issues are central to the concept of equipoise. Baker et al., [43] highlight how women must believe equipoise exists before considering to participate in research. It was evident in our study that some women did not believe equipoise existed. Most felt products worked. Distrust of equipoise has also been highlighted by Oude Rengerink et al., [20] in their qualitative case control study on barriers and motivators for pregnant women participating in Dutch trials. In addition, participants in our study thought that using any product was better than not doing anything and some also thought that the intervention product in the pilot trial was not as good as some of their known products despite not having evidence to support this view. Perhaps these participants needed clearer information on the study, as highlighted by Baker et al., [43]. Baker et al., [43] suggest that if study information is sufficient for trial participants there should be no doubt in relation to equipoise. They also advocate involving women in the development of study information to ensure it is presented in an appropriate manner. However, in our case it is unclear if this would have changed women’s decision not to participate.

Both randomisation and equipoise or uncertainty are integral to randomised trials. Randomisation is the foundation for ‘...testing the statistical significance of differences...’ between the intervention and control groups in relation to the trial outcomes [44] (p.155) while uncertainty, equipoise or ‘clinical equipoise’ in the context of the ‘community’ refers to the principle that there is no agreement as to which treatment is best [45]. Furthermore, randomisation is considered to be the most ethical means of assigning participants to study groups in the presence of clinical equipoise [46]. Notwithstanding this, these concepts have proven to be problematic for participants [47]. Research has shown that participants might either not understand or remember the information given to them on both concepts [47]. While acknowledging the complexity of the task of explaining randomisation, Robinson et al., [47] suggest focusing on its scientific advantages through a clear understandable explanation, while also helping potential participants to consider how the trial will advance knowledge on the topic. More recently, and related to the clear explanation suggested by Robinson et al., [47], is the need for some communications skills’ training for clinicians recruiting to clinical trials [45]. This recommendation arose from a study on how clinicians communicated equipoise to potential participants during recruitment and included both interviews with the clinicians and audio recording of the recruitment meeting. These findings may be beneficial in the context of future trials on the prevention of striae gravidarum.

Support for the second category identified under this theme: ‘trial requirements should fit with my routine’ is also evident in the trial by Ballantyne et al., [39] (p.480). In that study, the participants identified the main burdens to participating in research as being associated with the ‘inconvenience and time commitment’ involved. They wanted to know how the ‘time commitments ... would fit into their schedule’. This was consistent with our data. Furthermore, Ballantyne et al., [39] report how some women had said they had declined to participate in other studies during pregnancy where greater time commitment was involved. Similarly, time was a barrier in the qualitative study by Ayoub et al., [48] on recruitment and participation of pregnant women in research. They highlighted how having a balance between participation and other life demands was central in the decision to participate in research. Some of our participants said they were unable ‘to commit’ to the trial requirements, which resonates with Baker et al., [43] (p.65) who said ‘...issues, such as self-commitment..’ are central to deciding to participate in a study. The linkage between non or partial compliance with trial requirements and study results can be seen in other studies. For example, Ballantyne et al., [39] (p.479), report how the participants cared about the study outcome, which included being interested in the results and hoping that the ‘...study would show meaningful results’.

In relation to diary related issues and how smart phone based diaries were suggested by some participants to ensure compliance, caution is advised by Laughland & Kvavilashvili [49] (p.552) from their recent studies in the UK. They compared participant owned smart phone based diaries and paper diaries in three studies designed to determine if smart phones were associated with greater compliance and a higher number of entries compared to the paper diary. They found that those using their smart phone were compliant because they had diaries with them continually and completed their diary entry sooner; however, in all three studies ‘significantly fewer memory events were recorded in smart phones than paper diaries...’. They offer some suggestions for overcoming this challenge around sending reminders to participants using their smart phone and conclude how ‘participant-owned smartphone diaries will become the standard tool’ [49] (p.561).

In our study, some participants wished to see a better known product being investigated while some had a preference for organic products. We were using a common baby oil considered to be a hypoallergenic Paraben-free product. Baby oil tends to be chosen by a smaller proportion of women to prevent stretch marks in pregnancy but nevertheless continues to be some women’s choice [1, 8, 15] and, as with other products used by women, there is a lack of strong evidence of effectiveness from high quality trials [7] to support its use. Our participants also mentioned a product that has research to support its effectiveness, which may relate to some misunderstanding of randomised trials and the concept of equipoise as highlighted above or indeed the influence of product marketing. The suggestion for use of more organic or natural products is not surprising due to their attraction for pregnant women who increasingly request advice on natural remedies for the minor disorders of pregnancy [50].

Finally, we interpreted from the data that incentives may be useful but not a major determining factor in participation in a future study. Women’s decision to participate in research is often related to their belief in contributing to scientific inquiry [20, 39, 43], wanting to help others or the chance of a better outcome for their own pregnancy [37, 43]. For example, participants in the ORACLE study were influenced by the possibility of delaying pre-term labour [37]. While participants in the MAGPIE trial of prophylactic anti-convulsants for severe pre-eclampsia reported that participation was a means to getting the pharmacological treatment, which was otherwise unavailable to them and which they had been advised would prevent a seizure [51]. Some studies also refer to the concept of altruism [43, 48], which is often related to contributing to scientific inquiry and benefitting society and themselves [48].

Participants in our study were not over burdened by requests to participate in other research studies since becoming pregnant. We are not aware of any evidence on research demands on women during pregnancy but Close et al., [18] report how participation in another study was an exclusion criteria in their study due to the extra time and travel expense burden. Not being asked to join other studies was most likely related to the early stage of their pregnancy for participants in our study. Timing and how participants are approached are also important [19, 43]. Timing in particular has been identified as a factor for non-participation, as has being given ‘an overwhelming amount of information’ [20] (p.8). Information overload has relevance for our study due to recruitment to the pilot trial being at the booking visit when much other information is given to women, but this did not seem to be an issue in our study overall perhaps because the waiting times at the clinic allowed women adequate time to discuss the trial. Nevertheless, in other studies women have reported how a ‘conducive environment’ is important during study recruitment [43] (p.63) and antenatal clinics can be busy areas.

Our study has some limitations. The participants were from one geographical area and only included English speaking women. In addition, all participants had been invited to participate in a single trial investigating the effectiveness of a moisturising oil (Baby oil) compared to no treatment for the prevention and reduction in severity of striae gravidarum. However, a strength is that factors identified relate to actual decisions not to participate in a real trial rather than a hypothetical trial or trial scenario, and, as such, our findings may inform recruitment strategies in other similar trials. This focus on a real, rather than a hypothetical trial is in keeping with the plans for the Cochrane review of recruitment strategies for randomised trials which will be limited to real trials only in future updates [52].

## Conclusion

This qualitative descriptive study contributes to the existing body of evidence on striae gravidarum and offers insight into the factors influencing recruitment to the pilot trial on their prevention. It highlights how stretch mark prevention in pregnancy is important to most women and most take preventative action. Product choice is important and while there is a lack of strong evidence in relation to the effects of most of the products women are using, women do not seem to be influenced by that. In addition, participants in our study thought that using any product was better than not doing anything and some also thought that the intervention product proposed for the pilot trial was not as good as some of their known products, despite not having evidence to support this opinion.

Key points arising from this study for future striae gravidarum prevention trials is that a trial involving a well-known intervention product may be more likely to be accepted by women and may promote recruitment, while trial requirements need not be too burdensome on women. Finally, our study suggests that further work is required to overcome the non-acceptability of randomisation and to convey equipoise or uncertainty during trial recruitment, although our discussion section includes some suggestions for future trials on the prevention of striae gravidarum.

## Data Availability

The data analysed during the current study are available from the corresponding author on reasonable request.
